# Comparison of myocardial T1 mapping techniques at 1.5T to detect interstitial fibrosis in patients with orthotopic cardiac transplant

**DOI:** 10.1186/1532-429X-16-S1-P391

**Published:** 2014-01-16

**Authors:** Patrizia Pedrotti, Gabriella Masciocco, Giuseppina Quattrocchi, Angela Milazzo, Maria Frigerio, Paola Campadello, Alberto Roghi, Ornella Rimoldi

**Affiliations:** 1 IBFM, CNR, Segrate, Italy; 2 Dipartimento Cardiologico A. De Gasperis, Ospedale Niguarda Cà Granda, Milan, Italy; 3 Universita Vita Salute, San Raffaele, Milan, Italy

## Background

Apoptotic death of interstitial cells and myocytes is a detrimental effect of the interstitial inflammatory infiltrate accompanying heart transplant rejection. This inflammatory reaction is followed by both replacement fibrosis and interstitial fibrosis which in the long term can impair diastolic and systolic ventricular (LV) function. Cardiac magnetic resonance (CMR) with gadolinium quantifies replacement fibrosis and new sequences with T1 mapping are proposed for the quantification of more elusive interstitial fibrosis. We aimed at determining the optimal T1 mapping approach to assess characteristic tissue composition in these patients.

## Methods

We studied 60 patients who underwent orthotopic heart transplant (HTx) and were free of active rejection, mean time from Tx 79 ± 79 mo (range 6 ÷307) age 47 ± 13 and 10 Normals (N) 39 ± 11 p = ns. Standard volumes and LGE-CMR scans were carried out on a 1.5-T scanner (Siemens, Erlangen, Germany), with full myocardial coverage. The IR LGE images were acquired after intravenous Gadobutrol (0.15 mmol/kg) in identical short-axis planes to cine. MOLLI T1 maps (Messroghli, 2007) were generated from 3 short axis slices at Base, Mid and Apex and acquired pre and 15 minutes post contrast, parameters were TR = 2.5 msec, TE = 1.1 msec, Flip Angle 35°, Voxel size 2.2×1.4×6 mm, GRAPPA = 2. Regions of interest (ROIs) were defined defined according to AHA avoiding areas with coarse LGE or artifacts. Extracellular volume (ECV) was derived from T1-maps acquired pre- and post-contrast calibrated by blood hematocrit. Data are mean ± SD

## Results

**Figure 1:**
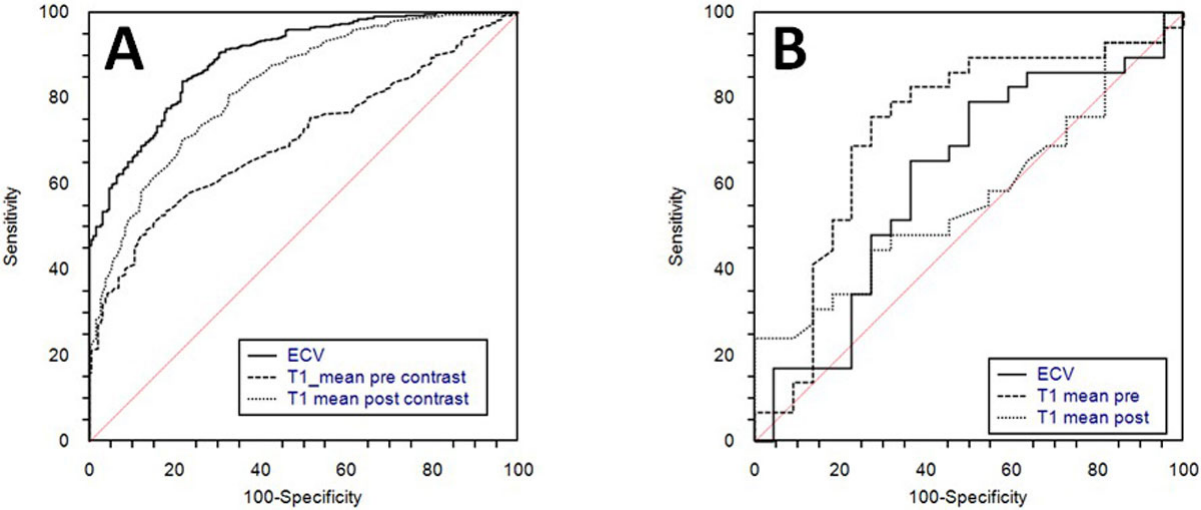


Systolic function was similar in both groups HTx 64 ± 11 vs N 68 ± 5 p = 0.32, LV Mass Index was slightly higher in HTx 75 ± 14 vs 69 ± 16 N p = 0.2. In HTx native pre -contrast T1 was 1007 ± 75 vs (N) 957 ± 44 msec p < 0.001, T1 post-contrast was 400 ± 47 msec vs (N) 455 ± 35 msec p < 0.001, ECV was 39.4 ± 4% vs 33.3 ± 3%(N) p < 0.001. ECV had the best Sensitivity(Se) 84% and Specificity(Sp) 78% at ROC analysis (cut-off 35.4%), for identifying HTx patients from N (Figure 1A). Whereas within Htx population T1 precontrast was more accurate to identify previous systemic CMV infection Se 77% and Sp 75% (cut-off 993 msec) (Figure 1B). T1 Pre was significantly higher in HTx with left ventricular hypertrophy P = 0.02. ECV, but not T1 pre or post, was inversely correlated to IVRT (msec) at echo p < 0.003.

## Conclusions

HTx patients with no active rejection show a significant increase of ECV and T1 relaxation time, an indication of increased interstitial fibrosis. In this population ECV seems to better identify deposition of collagen, whereas non contrast T1 mapping is also influenced by the presence of tissue inflammation. These preliminary findings need further confirmation in large scale studies that will assess both the diagnostic and prognostic values of T1 mapping derived parameters

## Funding

CNR.

